# Supplementary material for: Pathophysiological Significance of Neutrophilic Transfer RNA-Derived Small RNAs in Asymptomatic Moyamoya Disease
Source: Cells. 2021 May 1;10(5):1086. doi: 10.3390/cells10051086 (PMC8147334; doi:10.3390/cells10051086)
Supplement: Supplementary file 1 [file cells-10-01086-s001.zip › cells-1149161 3rd supplementary materials/Data Supplements/Data Supplement V.zip!/Data supplement 6-mRNAs targets of tsRNAs/tsRNA-Glu-CTC-003/Diagrams/tRF-Glu-CTC-003_vs_ENST00000642017(PNPO).pdf]

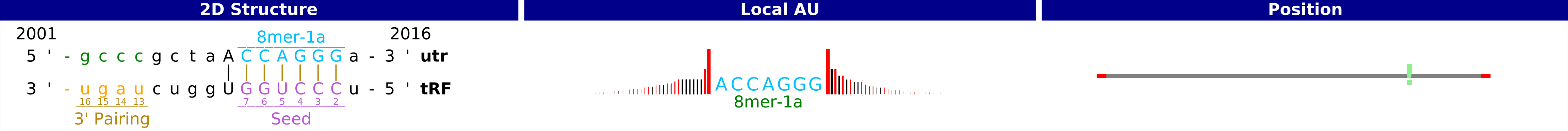
2D Structure
Local AU
Position
2001
2016
8mer-1a
5 ' - g c c c g c t a A C C A G G G a - 3 ' utr
3 ' - u g a u c u g g U G G U C C C u - 5 ' tRF
16 15 14 13
7 6 5 4 3 2
3' Pairing
Seed
ACCAGGG
8mer-1a
